# Ionophore Toxin Maduramicin Produces Haff Disease-Like Rhabdomyolysis in a Mouse Model

**DOI:** 10.3390/ijerph17217882

**Published:** 2020-10-27

**Authors:** Xiuge Gao, Xinhao Song, Runan Zuo, Dan Yang, Chunlei Ji, Hui Ji, Lin Peng, Yawei Qiu, Dawei Guo, Shanxiang Jiang

**Affiliations:** 1 Joint International Research Laboratory of Animal Health and Food Safety, College of Veterinary Medicine, Nanjing Agricultural University, Nanjing 210095, China; vetgao@njau.edu.cn (X.G.); sxh19940207@163.com (X.S.); 2018207004@njau.edu.cn (R.Z.); 2017107005@njau.edu.cn (D.Y.); 2018107006@njau.edu.cn (C.J.); jihui@njau.edu.cn (H.J.); 2016207003@njau.edu.cn (L.P.); qiuyawei@njau.edu.cn (Y.Q.); gdawei0123@njau.edu.cn (D.G.); 2 Laboratory of Veterinary Pharmacology and Toxicology, College of Veterinary Medicine, Nanjing Agricultural University, Nanjing 210095, China

**Keywords:** maduramicin, Haff disease, crayfish, rhabdomyolysis, ICR mice, ionophore antibiotic

## Abstract

Maduramicin is a toxic ionophore antibiotic that is isolated from *Streptomyces*, frequently occurring in an aquatic environment. To understand the potential role of maduramicin in crayfish consumption related Haff disease, a mouse model was established in this study. Two exposure routes of maduramicin in the abdominal muscle and the hepatopancreas tissue homogenates of crayfish were given intragastrically to mice in different doses for seven days. Action changes, clinical symptoms, feed consumption, body weight, blood biochemistry, and histopathology examination of mice were observed and analyzed. In the natural exposure group, relatively low concentration of maduramicin in crayfish muscle and hepatopancreas had no obvious effects on mental state, body weight, blood biochemical indexes, or histologic appearance. However, in the artificial exposure group, with increasing concentrations, maduramicin in crayfish muscle and hepatopancreas homogenates both induced mental sluggishness and weight loss of mice. Blood biochemical examination showed that 3.5 mg·kg^−1^ and 7 mg·kg^−1^ maduramicin in crayfish tissue homogenates significantly increased levels of alanine aminotransferase (ALT), aspartate aminotransferase (AST), blood urea nitrogen (BUN), lactate dehydrogenase (LDH), and creatine kinase (CK). Additionally, histopathological examination showed that multiple organs were damaged by maduramicin, including degeneration of liver cells, shedding of renal epithelial cells, and disturbance and partial lysis of myocardial and skeletal muscle filaments in the mice. In summary, maduramicin may not cause Haff disease through contamination of the aquatic environment under normal conditions. Maduramicin can be used as a potential toxin tool to establish a rhabdomyolysis disease animal model for drug development.

## 1. Introduction

Haff disease is a rare syndrome of unexplained myalgia and rhabdomyolysis in a person who has ingested seafood from fresh or brackish water within the previous 24 h. It was first reported in 1924 near the Königsberger Haff shores along the Baltic coast in East Prussia [1]. Epidemiological studies indicate that patients of Haff disease had consumed cooked fish or shrimp, including crayfish (*Procambarus clarkii*), buffalo fish (*Ictiobus cyprinellus*), freshwater pomfret (*Colossoma brachypomus*), Atlantic salmon (*Salmo salar*), pike (*Esox species*), and boxfish (*Ostraciontidae* spp.), which were probably contaminated by a heat-stable toxin [2,3,4]. Since the initial identification of Haff disease, similar outbreaks have been reported in many countries. From 1934 to 1984, in Sweden, the Soviet Union, and some other European countries, outbreaks of Haff disease occurred by consumption of the same cooked fish species in the earliest reports, including burbot (*L.* *lota*), pike (*Esox* spp.), and freshwater eel (*A. anguilla*) [1,5,6]. The first reported case of Haff disease in the United States occurred in 1984 [7], and as of 2014, 29 cases of Haff disease have been totally reported after ingestion of buffalo fish, crayfish, and Atlantic salmon in the United States so far [2,8]. In 2008, 25 cases of Haff disease were confirmed in Brazil due to consumption of fried or roasted pacu silver dollars (*Mylossoma* spp.), tambaqui black finned colossoma (*Colossoma macropomum*), or pirapitinga freshwater pompano (*Piaractus brachypomus*) [9]. In Japan, the confirmed Haff disease cases followed the ingestion of coastal marine boxfish [3]. In China, the first six cases of Haff disease outbreak were associated with eating crayfish, reported in Beijing in 2000 [10]. Subsequently, two outbreaks involving 54 cases in Lianzhou in 2009, and 23 cases in Nanjing in 2010, were reported [11,12]. A crayfish-related Haff disease case complicated by multiple organ failure was reported in Shanghai in 2013 [13]. Four hundred and ninety-four patients of Haff disease were totally reported between 5 July and 29 August 2016 from 15 different hospitals in Nanjing, China [14]. Increasing reports of Haff disease have attracted much more attention from scientists, however, there are two principal questions that need to be solved urgently. The first is the definite cause of Haff disease, and the second is that a Haff disease animal model should be established, which will be helpful to uncover the secrets of this disease.

As an important anticoccidiosis drug for the control of broiler chicken coccidiosis and growth promotion, maduramicin, one of the polyether ionophore antibiotics that were isolated from *Streptomyces*, is extensively used in the poultry industry [15]. Due to the narrow safety range and high toxicity of maduramicin, poisoning accidents occur frequently worldwide in the target animal (broiler chicken) by overdose or abuse, and non-target animals (laying hen, turkey, sheep, cattle, swine, canines, and rabbits) by misuse or cross-contamination of feed, as well as human by mistake [16,17,18,19,20,21]. Severe cardiomyopathy and rhabdomyolysis were observed in most poisoning cases, suggesting cardiac muscle and skeletal muscle are major toxicity target organs of maduramicin [22]. Humans who accidentally ingest maduramicin often show severe chest pain, myalgia with rhabdomyolysis, and sometimes acute renal failure [18], which are consistent with the symptoms of Haff disease [1,23]. Drugs in the same class, such as monensin and salinomycin, also have been reported as toxins that induce severe rhabdomyolysis with elevated serum creatine kinase (CK) levels and subsequent renal dysfunction in persons who accidentally ingest overdose amounts of ionophore antibiotics [24,25,26]. These intoxication cases induced by maduramicin or other ionophoric compounds illustrated that a serious health risk may be posed to the public under some extreme conditions.

Maduramicin was approved as an additive for control coccidiosis and growth promotion of broiler chickens at one day old during the whole period of growth. During the summer and autumn seasons, a large amount of maduramicin is widely and heavily used in the poultry production industry [27]. As maduramicin is poorly absorbed in the intestines of broiler chickens, the majority is excreted unchanged in chicken feces. In the broiler feces, 2.5–6.1 mg·kg^−1^ of maduramicin has been detected [16]. Considering that chicken waste application in land as a supplement to fertilizer is a common practice in many countries, there is rising international concern about the potential risk of veterinary drug residues in several environmental matrices [28,29]. In China, there is a typical method of using the feces and waste products of livestock discharging into the reservoir as aquaculture feed. Through these two exposure routes, large amounts of maduramicin was introduced into the environment. In Galicia, northern Spain, maduramicin and other ionophores (0.5 to 58.4 ng/L) were detected in the surface water that was collected from wastewater treatment plant fields [30]. Similarly, three ionophores, monensin, salinomycin, and narasin, were found in sediments at much higher concentrations than in surface water in northern Colorado in America [31]. Occurrence of ionophoric monensin (94 to 1077 µg/L) was detected in beef runoff pond water samples in northern Colorado, USA [32]. In addition, predicted environmental concentrations of four ionophore antibiotics (monensin, salinomycin, narasin, and lasalocid) in all environmental compartments and measured environmental concentrations in sediments are above predicted no-effect concentrations, so ionophores might pose an environmental risk [33]. Consumption of ionophore-contaminated aquatic products may threaten human health via food chain transfer.

Crayfish (*Procambarus clarkia*) consumption is very popular in central and south China during July and August. This correlates with maduramicin application in poultry production and the peak of crayfish-related Haff disease cases during the year. In Nanjing, people consume 60–80 tons of crayfish per day from June to September each year [34]. Crayfish ingestion accounted for almost all of the outbreaks of Haff disease in China since 2000, and more than 600 cases have been confirmed in total [4,12,14,35,36,37]. In this respect, crayfish was suspected to be contaminated by heat-stable toxins, which can be concentrated in fish edible tissues with resulting rhabdomyolysis. Although experimental studies have demonstrated toxic buffalo fish in USA and toxic pomfrets in China, and intoxicated mice associated with rhabdomyolysis [7,11], attempts to isolate and identify the toxin have so far failed. With increases in tourism and the global seafood trade and consumption, the geographical distribution and incidence of Haff disease are expected to increase. The responsible toxin for Haff disease needs to be identified for reducing unexplained health risk by consumption of fish products.

Based on crayfish-associated Haff disease and the fact that ionophore compounds induce rhabdomyolysis in humans and other animals, we hypothesized that the most toxic ionophore, maduramicin, may recapitulate Haff disease-like rhabdomyolysis in a mouse model. This study was performed to investigate the potential role of maduramicin in the induction of crayfish-related Haff disease by using a mouse model under two different exposure routes, and tries to establish a Haff disease animal model by analyzing clinical alterations.

## 2. Materials and Methods

### 2.1. Chemicals and Reagents

Maduramicin (CAS: 84878-61-5, batch number: 1,701,004, purity = 91.9%) was obtained from Zhejiang Esigma Biotechnology Co., Ltd. (Jiaxing, China) Phosphate buffer saline (PBS, P1031), giemsa staining solution (CAS: 51811-82-66, G1015), heparin sodium (CAS: 9041-08-1, H8060), and ethylene diamine tetra acetic acid disodium salt (EDTA-2Na, CAS: 139-33-3, E8030) were purchased from Solarbio Science & Technology Company (Beijing, China). Other analytical grade reagents used in the present study were obtained from Sinopharm Chemical Reagent Co., Ltd. (Shanghai, China).

### 2.2. Animals

To safeguard animal welfare, all of the methods performed in animals were carried out in strict accordance with the National Institutes of Health standards established in the Guidelines for the Care and Use of Experimental Animals. All experimental protocols in animals were approved by the Ethics Committee on Animal Experimentation of Nanjing Agricultural University. Adult male crayfish (body weight, 19–23 g) were purchased from Freshwater Fisheries Research Institute of Jiangsu Province (Nanjing, Jiangsu, China). Crayfish were acclimated in a plastic tank containing fully aerated, dechlorinated tap water (21 ± 2 °C) for 14 d in advance. Water quality characteristics were measured and recorded daily. The concentration of dissolved oxygen was higher than 6.0 mg·L^−1^, pH at 7.0, total ammonia at <1 mg/L, and water hardness (as CaCO_3_) at 116–121 mg·L^−1^. Crayfish were fed with drug-free commercial pellets (1% body weight each day), and excrement and dead crayfish bodies were removed in time. During the whole acclimation period, cultured water was renewed every two days, and the cumulative mortality of the crayfish did not exceed 5%.

Adult Institute of Cancer Research (ICR) mice (6–8 weeks, 18–22 g) were purchased from Nanjing Qinglongshan Animal Breeding Farm (Nanjing, China). Mice were housed in stainless steel cages in an air-conditioned room maintained at 24 ± 2 °C and 55 ± 5% humidity with a 12 h dark/light cycle. All mice were acclimated for 7 d prior to crayfish tissue exposure, and they were allowed free access to a normal diet and water.

### 2.3. Crayfish Exposure to Maduramicin

To simulate the natural exposure pathway of maduramicin to crayfish, 1/100, 1/20, and 1/10 LC_50_ of maduramicin on crayfish (0.7 mg·L^−1^, 3.5 mg·L^−1^, and 7 mg·L^−1^) were used in exposure treatment to obtain maduramicin contaminated crayfish tissues that could be used for subsequent mice intragastric administration. Maduramicin (0.007, 0.035, and 0.070 g) was dissolved in 1.5 mL of ethanol and diluted in 10 L of fully aerated dechlorinated tap water. As a vehicle control, 1.5 mL of ethanol was dissolved in 10 L of cultured water in the inert plastic tanks. Twenty crayfish per tank were used in the whole trial. All of the crayfish were exposed to maduramicin and solvent control for 72 h based on a preliminary study (unpublished data). After 72 h of exposure, the concentrations of maduramicin in the hepatopancreas of different treatment groups were 89.5, 406.5, and 756.7 μg·kg^−1^ and in abdominal muscles were 12.3, 83.5, and 135.5 μg·kg^−1^, respectively (unpublished data). Feeding conditions in the whole exposure study were in accordance with the acclimation process of crayfish.

### 2.4. Crayfish Sample Collection and Preparation

After maduramicin exposure, the hepatopancreas and abdominal muscles (without the intestine cord) of all crayfish were collected and stored at −80 °C. The obtained crayfish tissues were divided into the natural exposure group and artificial exposure group. To prepare tissue homogenates in the natural exposure group, in brief, hepatopancreas and muscles of crayfish with different concentrations of maduramicin were homogenized with physiological saline in a ratio of 1:1. The concentrations of maduramicin in different tissues were converted to the doses for mice gavage, 0.00025, 0.00167, and 0.00271 mg·kg^−1^ in muscles and 0.00179, 0.00813, and 0.01513 mg·kg^−1^ in hepatopancreas. In the artificial exposure group, the other 60 negative hepatopancreas and muscles were separately homogenized with maduramicin solution in a ratio of 1:1 to prepare homogenates, making the gavage doses of maduramicin in the homogenates 0.7 mg·kg^−1^, 3.5 mg·kg^−1^, and 7 mg·kg^−1^ that corresponded to 1/50, 1/10, and 1/5 LD_50_ of maduramicin on mice. The hepatopancreas and abdominal muscles of the remaining crayfish were used as a vehicle control and were homogenized with normal saline at a 1:1 ratio. All homogenates were prepared and stored at 4 °C prior to mice gavage on the same day.

### 2.5. Mice Gavage Test

One hundred and fifty ICR mice were randomly divided into fifteen groups. Three groups were the normal saline group, negative homogenate of crayfish hepatopancreas, and muscle group. Natural exposure group: 30 mice were divided into three groups and were administered crayfish muscle homogenate with different doses of maduramicin (0.00025, 0.00167, and 0.00271 mg·kg^−1^), and the other 30 mice were administered crayfish hepatopancreas with different doses of maduramicin (0.00179, 0.00813, and 0.01513 mg·kg^−1^). Artificial administration group: 30 mice were divided into three groups and orally administered the homogenates of crayfish abdominal muscle with maduramicin (0.7, 3.5, and 7 mg·kg^−1^), and the other 30 mice were administered the homogenates of crayfish hepatopancreas with different doses of maduramicin (0.7, 3.5, and 7 mg·kg^−1^). All mice of the fifteen groups were orally administered normal saline, negative crayfish homogenates, or maduramicin contaminated crayfish tissue homogenates (0.2 mL·10 g^−1^ bw·d^−1^) once a day for seven consecutive days.

### 2.6. Mice Sample Collection

During the whole animal experiment period, the general clinical observation of mice was performed once a day at eight in the morning. The weight of each mouse and the food consumption of each group were recorded every day. At day seven, mice were fasted for 4 h before sample collection. Blood samples were collected via orbit venous sinus into centrifuge tubes and centrifuged at 3500 r·min^−1^ for 10 min, and the obtained serum was stored at −20 °C for subsequent blood biochemistry analysis. Selected organs, including the heart, liver, kidney, and muscle, were collected (the blank control group, tissue control group, natural exposure group: high dose group, artificial administration group: high dose group), dissected, and fixed in neutral buffered formalin solution (10%, *V*/*V*) for further histopathology analysis.

### 2.7. Biochemical Analysis

Biochemical parameters lactate dehydrogenase (LDH), alkaline phosphatase (ALP), aspartate aminotransferase (AST), alanine aminotransferase (ALT), blood urea nitrogen (BUN), and creatine kinase (CK) were determined using commercial assay kits provided by Nanjing Jiancheng Bioengineering Institute (Nanjing, China). The detailed protocols were carried out according to the manufacturer’s instructions.

### 2.8. Histopathology Examination

The fixed tissue samples were embedded in paraffin wax, sectioned into two slides of 5 μm thickness, subsequently dehydrated in a series of graded ethanol, and stained with hematoxylin and eosin (H&E). After that, each tissue section was observed and photographed under a light microscope (OLYMPUS, CX23) with a digital camera for histopathology analysis.

### 2.9. Data Analysis

Results were presented as mean ± SD (standard deviation). Statistical analysis of the data was performed using one-way ANOVA, followed by an LSD (least significant difference) multiple comparison test by SPSS (version 22.0, IBM Corporation, Armonk, NY, USA). The value of *p* < 0.05 was considered as statistically significant.

## 3. Results

### 3.1. General Observation

To understand the effects of maduramicin on clinical symptoms of mice, we observed the general performance of all tested mice. Compared to the negative control group, the mice in the natural exposure group exhibited good activity and smooth fur. However, the mice in the artificial exposure group that were administered with a high dose of maduramicin (7 mg·kg^−1^) showed low activity, listlessness, limb weakness, thinness, and reduced spontaneous activity. Several mice in the artificial exposure group that were administered with a medium dose of maduramicin (3.5 mg·kg^−1^) exhibited toxic symptoms, such as mental depression, slow response, and thinness. The mice administered with the low dose of maduramicin (0.7 mg·kg^−1^) appeared to have normal activity and mentality compared to the negative control group.

### 3.2. Effects of Crayfish Tissues with Maduramicin on the Body Weight of Mice

As shown in Table 1, compared to the negative control, maduramicin-contaminated crayfish muscle in the natural exposure group had no effects on body weight and weight gain of mice. In contrast, the mice treated with maduramicin-contaminated muscle in the artificial exposure group showed significant weight loss in a dose-dependent manner (*p* < 0.05). As shown in Table 2, maduramicin-contaminated crayfish hepatopancreas in the natural exposure group showed no effects on the body weight of mice. The mice exposed to maduramicin-fortified crayfish hepatopancreas had significant weight loss compared to the control mice (*p* < 0.05).

### 3.3. Effects of Crayfish Tissues with Maduramicin on Feed Consumption of Mice

**Figure 1:**
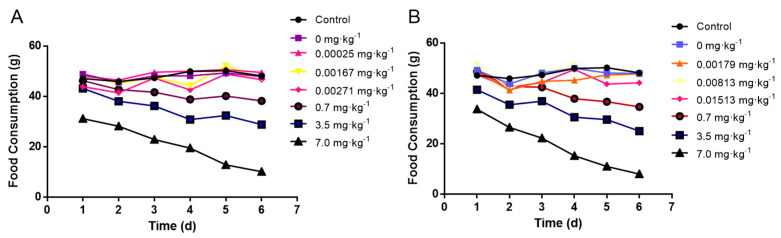
Effects of crayfish tissues with different concentrations of maduramicin on the feed consumption of mice. (**A**) Orally administered muscle homogenates containing different doses of maduramicin. All mice were orally administered normal saline, negative crayfish homogenates, or maduramicin contaminated crayfish tissue homogenates (0.2 mL·10 g^−1^ bw·d^−1^) once a day for seven consecutive days. Total feed consumption of each group was recorded daily. (**B**) Oral administration of hetapopancreas homogenates containing different doses of maduramicin. All mice were orally administered normal saline, negative crayfish homogenates, or maduramicin contaminated crayfish tissue homogenates (0.2 mL·10 g^−1^ bw·d^−1^) once a day for seven consecutive days. Total feed consumption of mice in one group were recorded once a day.

Changes in feed consumption during the whole period of the animal experiment were recorded and quantified in Figure 1. There was no significant difference in feed consumption between the negative control and the natural exposure group due to the low concentration of madudamicin in the contaminated crayfish muscle and hepatopancreas. In addition, the high dose of maduramicin in the contaminated crayfish tissues decreased the feed consumption of mice from day two to day seven. With the increase of maduramicin concentration (0.7 to 7 mg·kg^−1^), the feed intake of the mice was gradually reduced (Figure 1A,B).

### 3.4. Effects of Crayfish Muscle with Maduramicin on Blood Biochemistry of Mice

**Figure 2:**
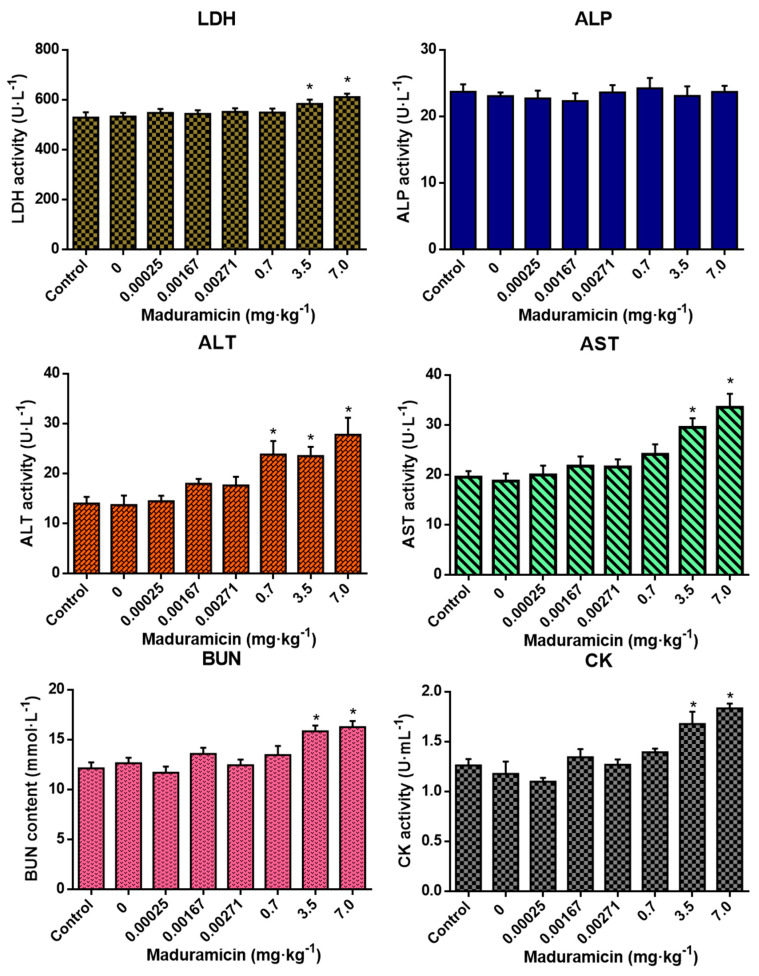
Effects of crayfish muscle with different doses of maduramicin on the blood biochemical indexes of mice. Mice were orally administered normal saline, negative crayfish muscle homogenates, or different doses of maduramicin in crayfish muscle homogenates from natural (0.00025 to 0.00271 mg·kg^−1^) and artificial (0.7 to 7 mg·kg^−1^) exposure pathways for seven days. After that, the serum of all treated mice was collected for blood biochemical analysis. The asterisks represent a significant difference between maduramicin treatment and the negative control group (n = 10, *, *p* < 0.05).

As shown in Figure 2, feeding with blank crayfish muscle homogenates showed no significant changes in all tested blood biochemical indexes of mice. Compared to the negative control, in the natural exposure group, maduramicin-contaminated crayfish muscle had no significant effects on the tested blood biochemical indexes. However, in the artificial exposure group, 0.7 mg·kg^−1^ of maduramicin in crayfish muscle significantly increased ALT activity (*p* < 0.05). When the concentration of maduramicin was elevated to 3.5 and 7 mg·kg^−1^, LDH, ALT, AST, BUN, and CK of mice were increased significantly (*p* < 0.05) compared to the negative control group.

### 3.5. Effect of Crayfish Hepatopancreas with Maduramicin on Blood Biochemistry of Mice

**Figure 3:**
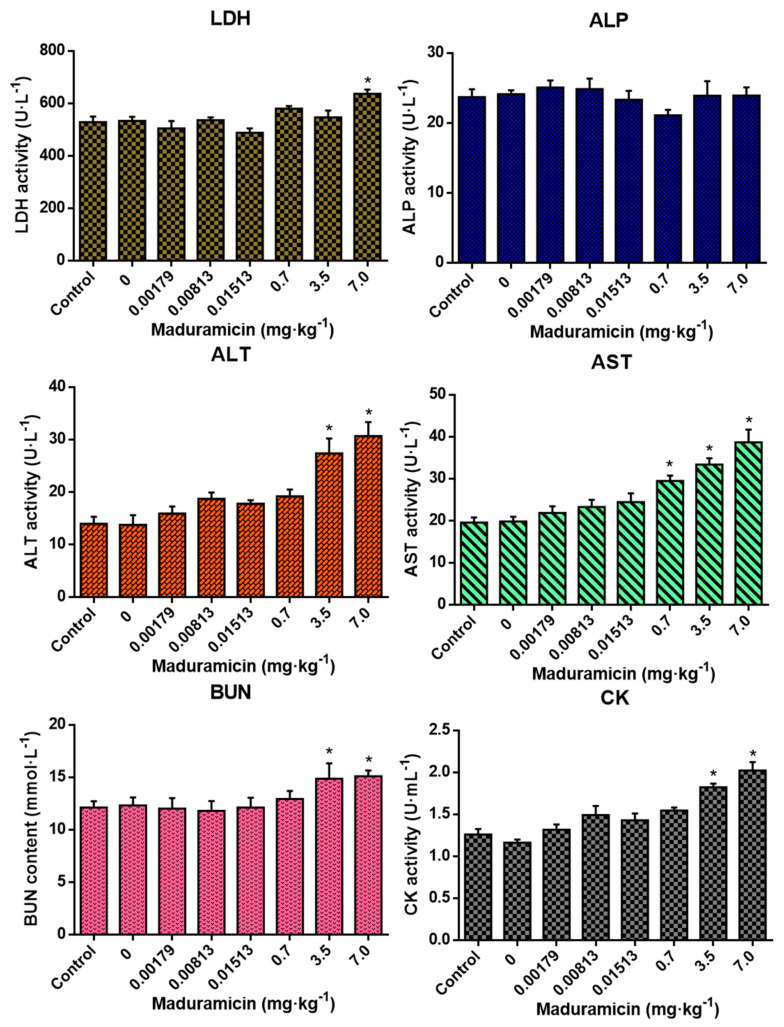
Effects of crayfish hepatopancreas with different doses of maduramicin on the blood biochemical indexes of mice. Mice were orally administered with normal saline, negative crayfish hepatopancreas homogenate, or different doses of maduramicin in crayfish hepatopancreas homogenate that originated from the natural (0.00179 to 0.01513 mg·kg^−1^) and the artificial (0.7 to 7 mg·kg^−1^) exposure pathways for seven days. After that, the serum of all treated mice was collected for blood biochemical analysis. The asterisks represent a significant difference between maduramicin treatment and the negative control group (n = 10, *, *p* < 0.05).

As shown in Figure 3, compared to the blank control, crayfish hepatopancreas without maduramicin had no effects on all tested blood biochemical indexes. In the natural exposure group, crayfish hepatopancreas with maduramicin showed no significant changes in all tested blood biochemical indexes (*p* > 0.05). However, when the dose of maduramicin was increased to 0.7 mg·kg^−1^ in the artificial exposure group, the AST activity of mice was elevated significantly (*p* < 0.05). In addition, ALT, AST, BUN, and CK of 3.5 mg·kg^−1^ and 7 mg·kg^−1^ maduramicin treated mice were significantly higher than that in the blank control (*p* < 0.05).

### 3.6. Histopathological Changes in Mice Exposed to Crayfish Muscle with Maduramicin

**Figure 4:**
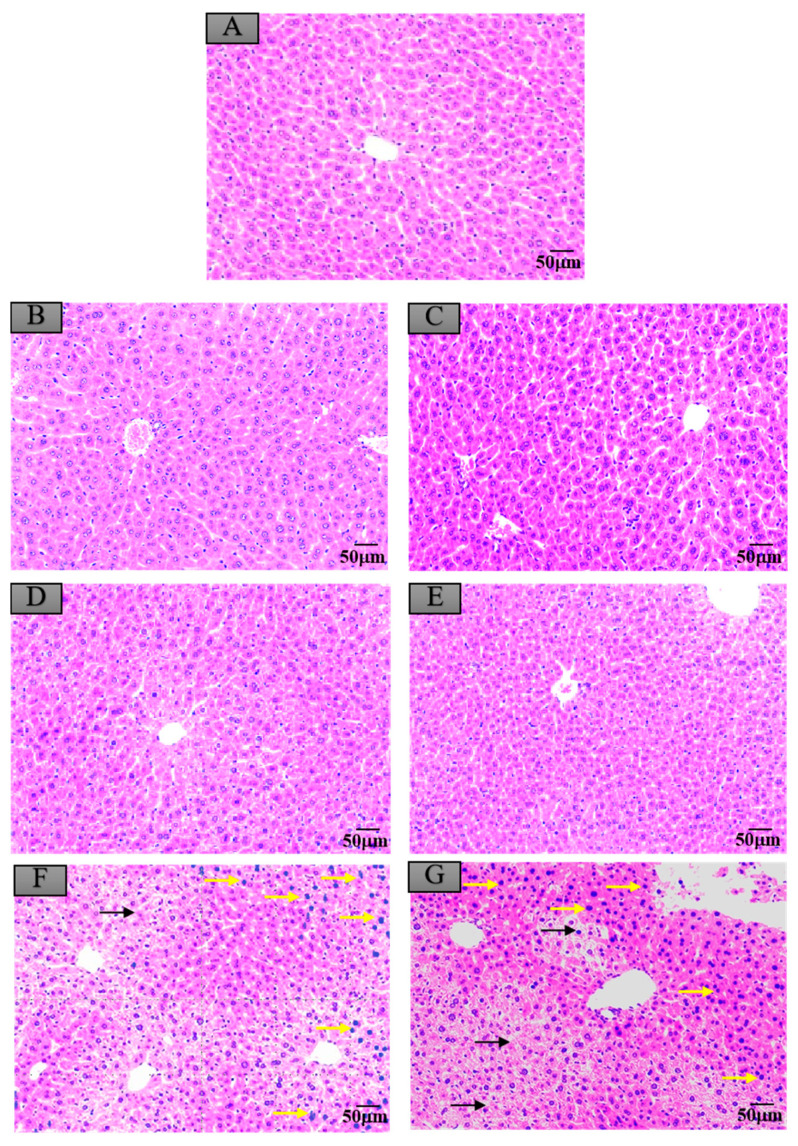
Histopathological changes of mice livers after exposure to maduramicin-contaminated crayfish tissues. Liver tissue samples were fixed in neutral buffered formalin solution (10%, *V*/*V*), embedded in paraffin wax, sectioned into sides of 5 µm thickness, subsequently dehydrated in a series of graded ethanol, and stained with hematoxylin and eosin (H&E) for microscopical observation under a light microscope equipped with a digital camera. (**A**) Negative control group. (**B**) Negative crayfish muscle control group. (**C**) Negative crayfish hepatopancreas control group. (**D**) Crayfish muscle homogenates with 0.00271 mg·kg^−1^ maduramicin. (**E**) Crayfish hepatopancreas homogenates with 0.01513 mg·kg^−1^ maduramicin. (**F**) Crayfish muscle homogenates with 7 mg·kg^−1^ maduramicin, showing vacuolar degeneration (black arrow) and nuclear condensation (yellow arrow). (**G**) Crayfish hepatopancreas homogenates with 7 mg·kg^−1^ maduramicin, indicating vacuolar degeneration (black arrow) and nuclear condensation (yellow arrow). The scale bar indicates 50 µm.

**Figure 5:**
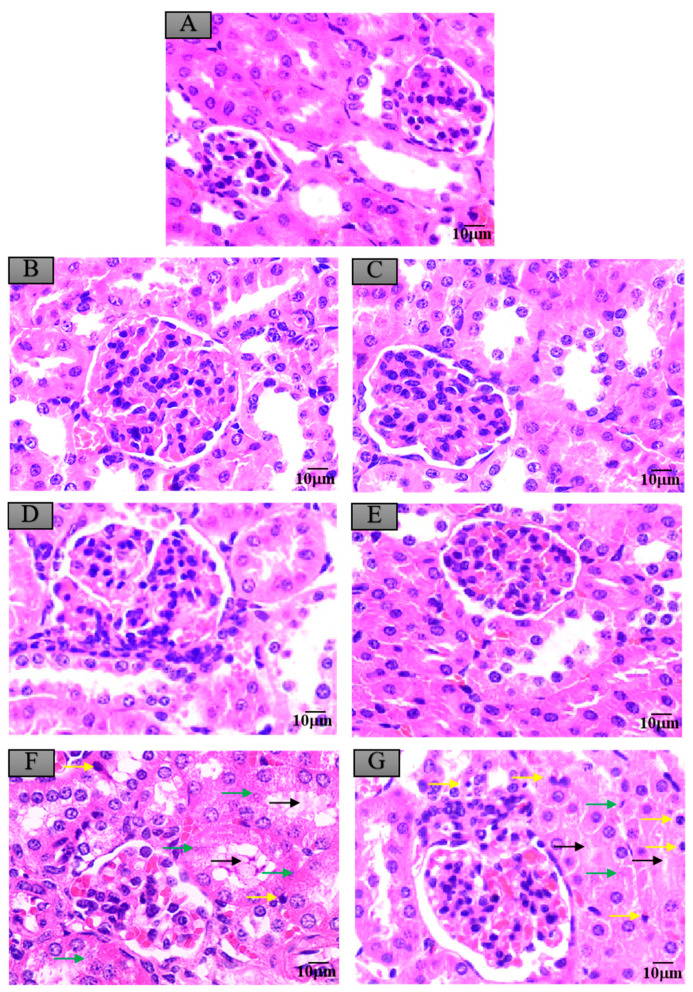
Histopathological changes of mice kidneys after exposure to maduramicin-contaminated crayfish tissues. Kidney tissue samples were fixed in neutral buffered formalin solution (10%, *V*/*V*), embedded in paraffin wax, sectioned into sides of 5 µm thickness, subsequently dehydrated in a series of graded ethanol, and stained with H&E for microscopical observation under a light microscope equipped with a digital camera. (**A**) Negative control group. (**B**) Negative crayfish muscle control group. (**C**) Negative hepatopancreas control group. (**D**) Crayfish muscle with 0.00271 mg·kg^−1^ maduramicin. (**E**) Crayfish hepatopancreas with 0.01513 mg·kg^−1^ maduramicin. (**F**) Crayfish muscle homogenates with 7 mg·kg^−1^ maduramicin, showing epithelial exfoliation (black arrow), nuclear condensation (yellow arrow), and nuclear fragmentation (green arrow). (**G**) Crayfish hepatopancreas homogenates with 7 mg·kg^−1^ maduramicin, showing epithelial exfoliation (black arrow), nuclear condensation (yellow arrow), and nuclear fragmentation (green arrow). The scale bar = 10 µm.

**Figure 6:**
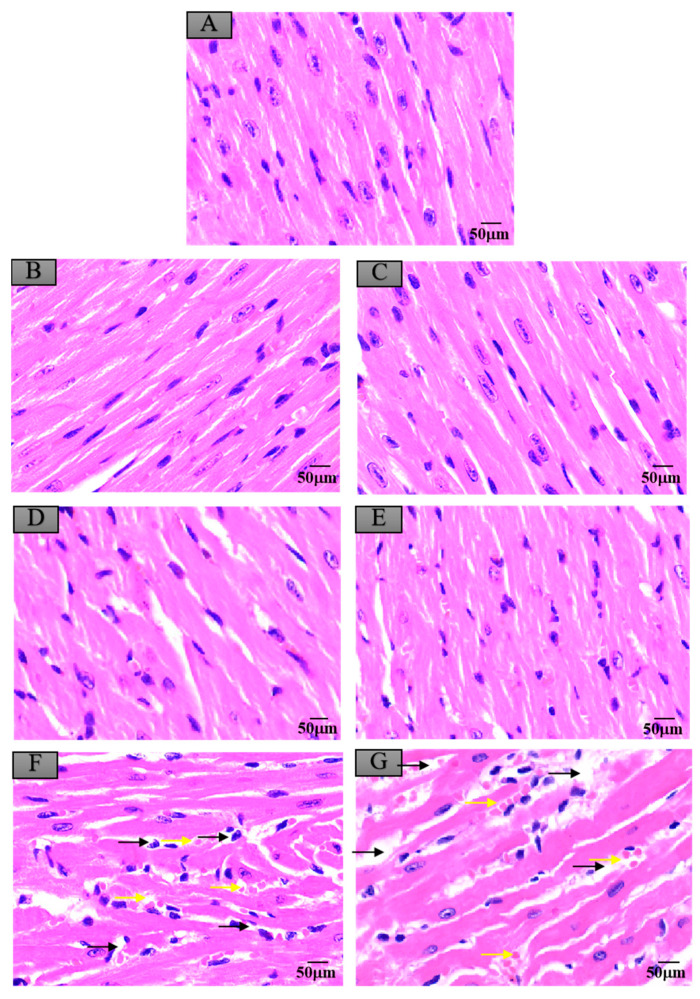
Histopathological changes of mice myocardium after exposure to maduramicin-contaminated crayfish. Myocardium tissue samples were fixed in neutral buffered formalin solution (10%, *V*/*V*), embedded in paraffin wax, sectioned into sides of 5 µm thickness, subsequently dehydrated in a series of graded ethanol, and stained with H&E for microscopical observation under a light microscope equipped with a digital camera. (**A**) Negative control group. (**B**) Negative crayfish muscle control group. (**C**) Negative crayfish hepatopancreas control group. (**D**) Crayfish muscle with 0.00271 mg·kg^−1^ maduramicin. (**E**) Crayfish hepatopancreas with 0.01513 mg·kg^−1^ maduramicin. (**F**) Crayfish muscle homogenates with 7 mg·kg^−1^ maduramicin, showing myocardial interstitial edema (yellow arrow) and fibrin exudation (black arrow). (**G**) Crayfish hepatopancreas homogenates with 7 mg·kg^−1^ maduramicin, showing fibrin exudation (black arrow) and myocardial interstitial edema (yellow arrow). The scale bar = 50 µm.

**Figure 7:**
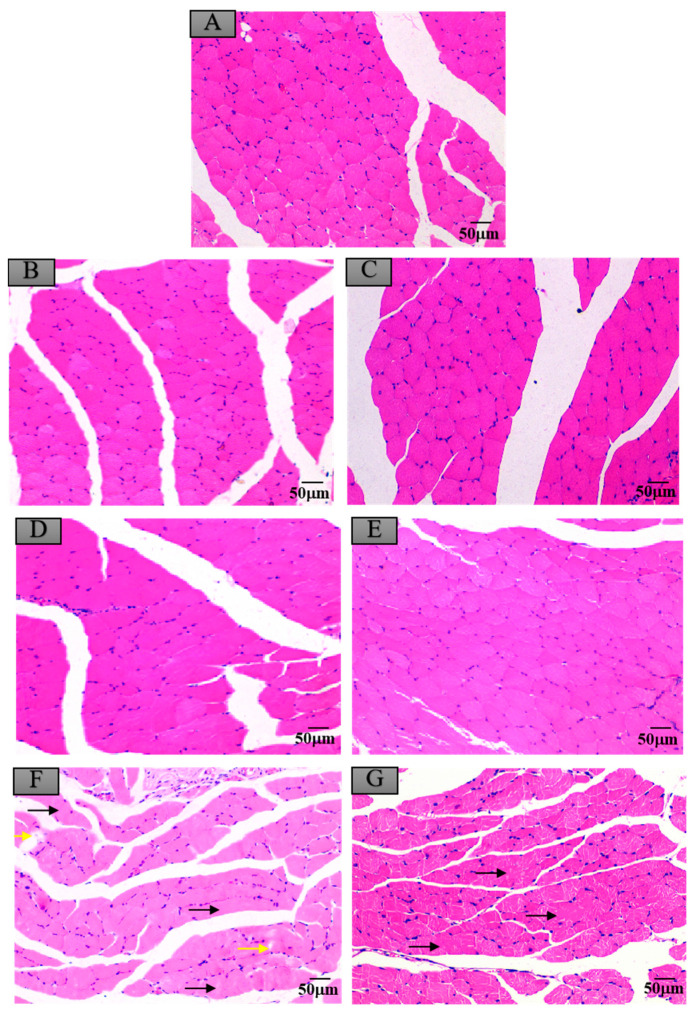
Histopathological changes of mice skeletal muscles after exposure to maduramicin-contaminated crayfish tissues. Skeletal muscle tissue samples were fixed in neutral buffered formalin solution (10%, *V*/*V*), embedded in paraffin wax, sectioned into sides of 5 µm thickness, subsequently dehydrated in a series of graded ethanol, and stained with H&E for microscopical observation under a light microscope equipped with a digital camera. (**A**) Negative control group. (**B**) Negative crayfish muscle control group. (**C**) Negative crayfish hepatopancreas control group. (**D**) Crayfish muscle homogenates with 0.00271 mg·kg^−1^ maduramicin. (**E**) Crayfish hepatopancreas homogenates with 0.01513 mg·kg^−1^ maduramicin. (**F**) Crayfish muscle homogenates with 7 mg·kg^−1^ maduramicin, showing muscle silk edema (yellow arrow) and rhabdomyolysis (black arrow). (**G**) Crayfish hepatopancreas homogenates with 7 mg·kg^−1^ maduramicin, showing rhabdomyolysis (black arrow). The scale bar = 50 µm.

Compared to the control group, as shown in Figure 4A,B,D, the livers of mice administered with negative crayfish muscle or contaminated with a low dose of maduramicin (0.00271 mg·kg^−1^) did not show pathological changes. On the contrary, in the artificial exposure group, crayfish muscle contaminated with the higher dose of maduramicin (7 mg·kg^−1^) caused severe liver injury in mice, exhibiting a deteriorated hepatic lobule, vacuolar degeneration (black arrow), and nuclear condensation (yellow arrow), as shown in Figure 4F. Histopathological alterations of the mouse kidney followed gavage of crayfish muscle homogenates with a low dose of maduramicin (0.003 mg·kg^−1^), and contamination showed intact tubular epithelial cells, normal glomerular size, and no degeneration (Figure 5A,B,D), which were consistent with the control group. A high dose of maduramicin (7 mg·kg^−1^) fortified in crayfish muscle homogenates induced severe degeneration of renal tubular epithelial cells, epithelial exfoliation (black arrow), nuclear condensation (yellow arrow), and nuclear fragmentation (green arrow) (Figure 5F). As for pathological analysis of mice hearts, as shown in Figure 6A,B,D, there were no observed abnormal changes between mice given crayfish muscle with a low dose of maduramicin (0.003 mg·kg^−1^) and the negative control, showing arranged muscle fibers and no inflammatory infiltration. A high dose of maduramicin (7 mg·kg^−1^) in crayfish muscle homogenates caused myocardial interstitial edema (yellow arrow) and fibrin exudation (black arrow) (Figure 6F). Compared to the skeletal muscle of mice in the negative control, mice given a low dose of maduramicin (0.003 mg·kg^−1^) in crayfish muscle homogenates did not show a change in muscle fiber arrangements or morphology of skeletal muscle cells (Figure 7A,B,D). However, a high dose of maduramicin (7 mg·kg^−1^) induced severe edema of muscle silk (yellow arrow) and rhabdomyolysis (black arrow in Figure 7F).

### 3.7. Histopathological Changes in Mice Exposed to Crayfish Hepatopancreas with Maduramicin

Compared to the control group, negative crayfish hepatopancreas homogenates or low dose maduramicin (0.015 mg·kg^−1^) had no effects on mice liver morphology (Figure 4A,C,E). In the artificial exposure group, a high dose of maduramicin (7 mg·kg^−1^) in crayfish hepatopancreas homogenates induced severe vacuolar degeneration (black arrow) and nuclear condensation (yellow arrow) of mice livers (Figure 4G). There were no observed pathological changes in kidneys of mice administered with negative crayfish hepatopancreas or with a low dose of maduramicin (0.015 mg·kg^−1^), compared to the blank control (Figure 5A,C,E). A high dose of maduramicin (7 mg·kg^−1^) in crayfish hepatopancreas homogenates in the artificial exposure group caused epithelial exfoliation (black arrow), nuclear condensation (yellow arrow), and nuclear fragmentation (green arrow) (Figure 5G). Furthermore, a low dose of maduramicin (0.015 mg·kg^−1^) in crayfish hepatopancreas homogenates showed no abnormal changes in mice hearts compared to those of the blank control group and the negative crayfish hepatopancreas group (Figure 6A,C,E). In the artificial exposure group, a high dose of maduramicin (7 mg·kg^−1^) in crayfish hepatopancreas homogenates induced serious pathological lesions, such as disordered and broken myofilaments of mice (Figure 6G). Similarly, mouse skeletal muscles treated by crayfish hepatopancreas homogenates with a low dose of maduramicin (0.015 mg·kg^−1^) showed no observed differences with normal muscle tissue (Figure 7A,C,E). In contrast, a high dose of maduramicin (7 mg·kg^−1^) in crayfish hepatopancreas homogenates caused serious damage of skeletal muscle, showing rhabdomyolysis (black arrow in Figure 7G).

## 4. Discussion

Since the outbreak of Haff disease in early 1965, studies of the risk factors have been conducted consistently. For the first time, Leschchenko et al. in the Soviet Union experimentally induced a condition mimicking Haff disease in mice and cats by feeding them fresh fish implicated in a Haff disease outbreak [2], proposing the scientific opinion that an unknown fat-soluble, heat-stable fresh and/or brackish water algal toxin bioconcentrated in the aquatic food chain was the cause [2]. After that, during an outbreak of 29 Haff disease cases in the US, the Food and Drug administration (FDA) and the Center of Disease Control analyzed the known aquatic toxins, including ciguatoxin, saxitoxin, brevetoxin, tetrodotoxin, palytoxin, domoic acid, okadaic acid, and two blue-green algal or cyanobacterial toxins, microcystin and nodularin, but, unfortunately, all of the tested samples were negative [1]. The Japanese experience with rhabdomyolysis following marine fish consumption ruled out marine palytoxin poisoning because of the negative serum and urine palytoxin hemolysis activity in these cases [38]. Based on the investigation, Japanese scientists speculated the existence of a heat-stable myotoxin, similar to palytoxin, which can be sometimes contaminated or accumulated in coastal boxfish, a common inducer of rhabdomyolysis in Japan [2,38]. On further investigation, in 2001, the U.S. FDA laboratory findings suggested that heavy metals, pesticides, herbicides, and freshwater and marine algal toxins were negative in suspected crayfish samples [2]. Chinese scientists also spent a lot of effort in discovering the suspected toxins that are responsible for crayfish consumption related Haff disease, however, attempts to isolate and identify the toxins have so far failed. In 2009~2010, during the outbreak of Haff disease in Guangdong Province, China, Huang Qiong et al. analyzed fish, water, and soil samples for microcystins, nine toxins, including scorpion toxin, chelating base, histamine, ractopamine, and several acid ions, and 26 heavy metals by performing multiple chemical analysis techniques. Nevertheless, all tested chemicals were negative or lower than the toxicity thresholds [11]. Chen Xiaofeng et al. demonstrated that rhabdomyolysis related toxins from crayfish were not an allergen or palytoxin [39]. Chen Yan et al. have conducted chemical analysis from crayfish and the blood and urine samples of two patients during the 2010 Nanjing outbreak of Haff disease, and more than 200 compounds with known relevance to rhabdomyolysis were screened, but unfortunately, no possible toxins were revealed [12]. Recently, Sun Guiju et al. also attempted to explore the chemical etiology in a large outbreak of Haff disease between 30 June 2016 and 29 August 2016 in Nanjing, China [14]. Chemical assessment of crayfish, river water, and sediment that might induce rhabdomyolysis were negative, including anticoccidiosis drugs, niclosamide, organophosphorus pesticides, and microcystins [14]. Up to date, worldwide, the chemical etiology of Haff disease remains unidentified. The suspicious compounds that might cause contaminated Haff disease-related seafood should be investigated.

As neglected in previous studies, a potential rhabdomyolysis inducer, maduramicin, which is produced by *Actinomadura madurae*, was suspected due to its characteristics of heat-stable hydrophobicity. Maduramicin is the most toxic ionophore antibiotic with a very narrow safety range, and often causes severe rhabdomyolysis in target/non-target animals and humans who consumed it by accident [18,19,27]. In China, more than eight cases of maduramicin intoxication have been reported during occupational or accidental exposure. In India, there were seven human cases of poisoning with maduramicin in porridge by mistake, and four patients unfortunately died after 48 h, with a varying degree of rhabdomyolysis [18]. Other drugs of the same ionophore class, such as monensin and salinomycin, have been reported to cause human intoxication with different levels of rhabdomyolysis in China and other countries [24,25,26]. In addition, extensive use of maduramicin and other ionophores in the livestock production industry increased the environmental release and posed potential risk to ecology safety and public human health in recent years [20,33]. Previous studies show the occurrence of maduramicin and ionophore antibiotics in manure, surface water, sediments, and many other environmental compartments [31,32,40,41,42]. As emerging contaminants, the predicted concentrations in all environmental compartments and measured environmental concentrations in sediments are higher than predicted no-effect concentrations, suggesting a potential environmental risk of ionophores [33]. Moreover, in consideration of maduramicin-induced rhabdomyolysis in a variety of animals and high sediment concentrations of ionophores, the major aquatic vector of Haff disease in China was crayfish, a bottom-feeder freshwater crustacean [12]. We hypothesized that the most toxic ionophore, maduramicin, might play an important role in cases of crayfish consumption associated Haff disease in China among the summer season. In this study, to demonstrate the potential role of maduramicin in crayfish related Haff disease, we attempted to elaborate a rhabdomyolysis mice model by intragastric administration of maduramicin, mimicking natural (low concentration) and artificial (high concentration) contaminated crayfish muscle and hepatopancreas homogenate. These two exposure routes of maduramicin were designed to demonstrate whether a low concentration of maduramicin in crayfish edible tissue causes Haff disease symptoms in mice, and whether an acute Haff disease mice model would be established by using high concentrations of maduramicin.

To mimic natural exposure of maduramicin-contaminated crayfish to mice, 1/10, 1/20, and 1/100 LD_50_ of maduramicin in mice were used as the exposure dose to crayfish, and the final doses of maduramicin were 0.00025, 0.00167, and 0.00271 mg·kg^−1^ in muscles and 0.00179, 0.00813, and 0.01513 mg·kg^−1^ in hepatopancreas of crayfish, respectively. After administration of the crayfish tissue homogenate containing a low concentration of maduramicin by gavage for seven days, there were no significant changes in body weight, food intake, or blood biochemical indexes. Pathological observation showed no obvious histological changes compared to the negative control. Based on these findings, we first demonstrated that maduramicin residue in crayfish will not cause symptoms of rhabdomyolysis in mice under normal exposure conditions, which is consistent with the opinions of the European Food Safety Authority (EFSA) that ionophores have no harmful effects on humans, animal health, or the environment [43]. Furthermore, to explore whether maduramicin causes rhabdomyolysis under extreme conditions, we attempted to establish a mouse model of Haff disease by carrying out the artificial exposure of mice to a high dose of maduramicin (0.7, 3.5 and 7 mg·kg^−1^). Laboratory examination of blood biochemical indicators are crucial to the determination of Haff disease [34]. In general, serum CK of patients shows a sharp increase, which is more than five times higher than the normal level [44]. ALT, AST, Mb, and LDH were also abnormally elevated in more than 80% of patients [23]. In severe cases, acute renal failure occurs, leading to elevated BUN indexes [23]. In this study, it was found that the indexes of CK, ALT, AST, LDH, and BUN in mice treated with 3.5 mg·kg^−1^ and 7 mg·kg^−1^ of maduramicin were significantly increased in a dose-dependent manner. ALT and AST activities were increased significantly by maduramicin treatment, presumably due to damage to the liver and myocardial tissues, which allowed the release of transaminases into the blood. Elevated LDH levels also suggested skeletal muscle or myocardial damage by maduramicin exposure. As the most important indicator to diagnose Haff disease, CK often elevated significantly along with rhabdomyolysis in most Haff disease cases [4,35,36]. In the present study, only 7 mg·kg^−1^ of maduramicin induced CK elevation about two times higher than the normal level, indicating that the laboratory diagnostic index of Haff disease is still unable to be reached just by maduramicin treatment in mice. However, it cannot be ruled out that in a complex aquatic environment, maduramicin may have a synergistic effect with other unknown exogenous substances. A rhabdomyolysis animal model has been established in previous studies by coadministration of statin and fibrate [45], and ciprofloxacin and atorvastatin [46], both with a sufficient increase of serum CK values in C57BL/6J mice. They also demonstrated that, compared to BALB/c and ICR mice, C57BL/6J mice showed the highest sensitivity to pioglitazone-induced skeletal muscle injury [45]. Although our study did not show sufficiently high CK levels in maduramicin treated mice, the additional histopathology findings show that skeletal muscle degeneration and necrosis to develop rhabdomyolysis similar to Haff disease were observed. In addition, liver damage and kidney injury of mice treated by a high dose of maduramicin were found. These findings support that a single maduramicin treatment may not be sufficient to induce the characteristic feature of crayfish-associated rhabdomyolysis of Haff disease. Combined use of maduramicin with other known rhabdomyolysis inducer drugs or compounds will be beneficial for development of a crayfish-related Haff disease animal model that is useful for studying the therapeutic approach of rhabdomyolysis-associated Haff disease.

## 5. Conclusions

In conclusion, as an environmental pollutant, maduramicin will not induce crayfish-related Haff disease in a mouse model under normal circumstances due to its low exposure concentration. In some extreme conditions, maduramicin residue with a high concentration in edible tissues of crayfish will cause significant damage of skeletal muscle, myocardial muscle, livers, and kidneys of mice, but will not reached the criterion of Haff disease. Base on maduramicin-contaminated crayfish consumption in the mice model, it is concluded that maduramicin is unlikely to induce Haff disease by a crayfish-transmitted food chain.

## Figures and Tables

**Table 1:** Effects of crayfish muscle with different concentrations of maduramicin on the body weight of mice.

Group	Dose (mg kg^−^^1^)	Initial Weight (g)	Final Weight (g)	Body Weight Gain (g)
Blank control group	0	24.46 ± 2.35	30.24 ± 4.73	5.78
Muscle control group	0	24.97 ± 1.62	30.29 ± 2.58	5.32
Natural exposure group	0.00025	24.35 ± 1.20	30.73 ± 3.16	6.38
	0.00167	24.39 ± 1.82	28.62 ± 2.03	4.23
	0.00271	24.15 ± 2.20	28.47 ± 3.59	4.32
Artificial exposure group	0.7	25.27 ± 1.36	28.11 ± 3.02 *	2.84
	3.5	24.42 ± 1.18	24.45 ± 2.59 *	0.03
	7.0	24.54 ± 1.56	21.40 ± 3.84 *	−3.14

Note: the asterisks represent a significant difference between maduramicin treatment and the control group at the same time point (*, *p* < 0.05).

**Table 2:** Effects of crayfish hepatopancreas with different concentrations of maduramicin on the body weight of mice.

Group	Dose (mg kg^−^^1^)	Initial Weight (g)	Final Weight (g)	Body Weight Gain (g)
Blank control group	0	24.46 ± 2.35	30.24 ± 4.73	5.78
Hepatopancreas control group	0	23.82 ± 1.12	29.10 ± 2.85	5.28
Natural exposure group	0.00179	24.70 ± 1.02	30.54 ± 3.52	5.84
	0.00813	24.14 ± 1.44	28.50 ± 3.23	4.36
	0.01513	23.79 ± 0.55	27.89 ± 3.29	4.10
Artificial exposure group	0.7	24.69 ± 1.49	26.62 ± 3.18 *	1.93
	3.5	24.38 ± 1.58	23.32 ± 3.52 *	−1.06
	7.0	24.87 ± 1.18	21.13 ± 1.04 *	−3.74

Note: the asterisks represent a significant difference between maduramicin treatment and the control group at the same time point (*, *p* < 0.05).
